# The genome sequence of the painted lady, *Vanessa cardui *Linnaeus 1758

**DOI:** 10.12688/wellcomeopenres.17358.1

**Published:** 2021-11-26

**Authors:** Konrad Lohse, Charlotte Wright, Gerard Talavera, Aurora García-Berro, Darwin Tree of Life Barcoding collective, Wellcome Sanger Institute Tree of Life programme, Wellcome Sanger Institute Scientific Operations: DNA Pipelines collective, Tree of Life Core Informatics collective, Darwin Tree of Life Consortium

**Affiliations:** 1 Institute of Evolutionary Biology, University of Edinburgh, Edingburgh, UK; 2 Tree of Life, Wellcome Sanger Institute, Cambridge, UK; 3 Institut Botànic de Barcelona (IBB, CSIC-Ajuntament de Barcelona), Barcelona, Spain

**Keywords:** Vanessa cardui, painted lady, genome sequence, chromosomal; Lepidoptera

## Abstract

We present a genome assembly from an individual female *Vanessa cardui *(the painted lady; Arthropoda; Insecta; Lepidoptera; Nymphalidae). The genome sequence is 425 megabases in span. The majority of the assembly is scaffolded into 32 chromosomal pseudomolecules, with the W and Z sex chromosome assembled. Gene annotation of this assembly on Ensembl has identified 12,821 protein coding genes.

## Species taxonomy

Eukaryota; Metazoa; Ecdysozoa; Arthropoda; Hexapoda; Insecta; Pterygota; Neoptera; Endopterygota; Lepidoptera; Glossata; Ditrysia; Papilionoidea; Nymphalidae; Nymphalinae; Vanessa; *Vanessa cardui* (Linnaeus, 1758) (NCBI:txid171605).

## Background

The painted lady, *Vanessa cardui*, is an extremely widespread butterfly, occurring on all continents except most of South America and Oceania ( Shields, 1992). The species undertakes long-distance multi-generational migrations each year ( Pollard *et al.*, 1998; Stefanescu *et al.*, 2013; Talavera *et al.*, 2018; Williams, 1970). It does not overwinter and is therefore engaged in a constant movement. In the Palaearctic, migrants are known to seasonally circulate between North Africa and Europe ( Pollard *et al.*, 1998; Stefanescu, 2011; Stefanescu *et al.*, 2013). Recent work has also revealed that autumn populations from Europe cross the Sahara Desert reaching tropical Africa ( Stefanescu *et al.*, 2016; Talavera & Vila, 2016). This journey, spanning over 4000 km, represents the longest single-leg migratory flight known in butterflies. The butterflies migrate back to Europe in spring, thus covering up to 14000 km in an annual cycle involving 8–10 generations in their Palaearctic-African range ( Menchetti *et al.*, 2019; Talavera *et al.*, 2018). The painted lady is found throughout the British Isles but abundance varies greatly between years. Larvae are polyphagous on a large variety of plant families, but most commonly feed on thistles ( *Cirsium* spp *.* and *Carduus* spp *.*) and mallows ( *Malva* spp.). The painted lady occurs in a wide range of biomes and environments spanning semi-deserts, grasslands, meadows, and mountains to suburban areas. It is listed as Least Concern in the IUCN Red List ( Walker & Coetzer, 2020). Studies of *V. cardui* have included thermoregulation ( Tsai *et al.*, 2020), adaptations to host plants ( Celorio-Mancera *et al.*, 2016), flight behaviour ( Gamberale-Stille *et al.*, 2019; Liu *et al.*, 2021) and movement ecology ( Suchan *et al.*, 2018). Genes involved in the development of the distinctive eyespots on the forewings and hindwings of *V. cardui* have been identified ( Mazo-Vargas *et al.*, 2017; Zhang & Reed, 2016; Zhang *et al.*, 2017). *V. cardui* has a karyotype of 31 chromosomes ( Lorkovic, 1941).

We note the recent publication of another high-quality genome assembly for *V. cardui* ( Zhang *et al.*, 2021). We hope that the sequence described here, generated as part of the Darwin Tree of Life project, will further contribute to the study of *V. cardui* as an emerging model for the genetics of migratory behavior, ecological genomics and developmental genetics.

## Genome sequence report

**Figure 1.**
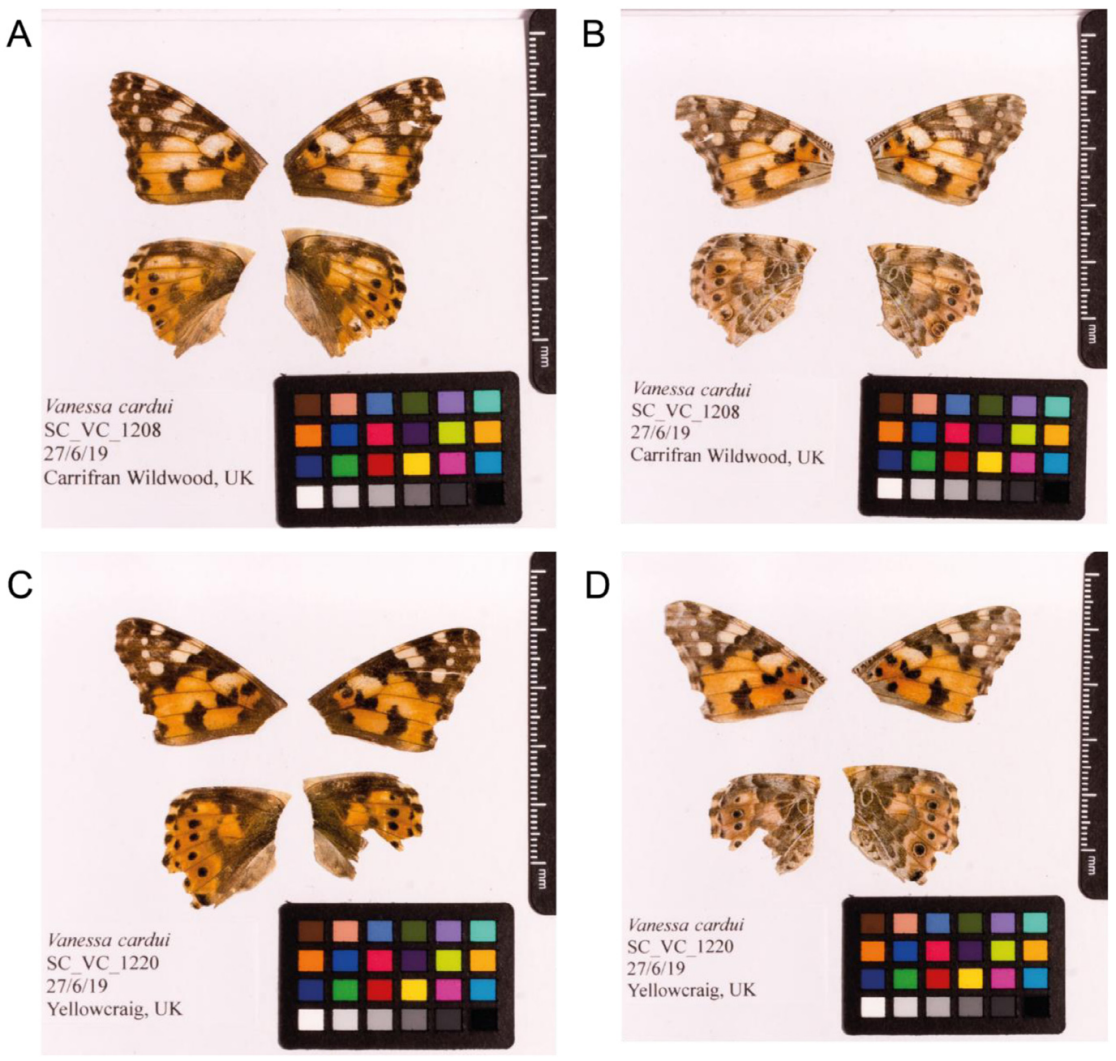
Fore and hind wings of *Vanessa cardui* specimens from which the genome was sequenced. ( **A**) Dorsal surface view of wings from specimen SC_VC_1208 (ilVanCard2) from Carrifran Wildwood, Scotland used to generate Pacific Biosciences and 10X genomics data. ( **B**) Ventral surface view of wings from specimen SC_VC_1208 (ilVanCard2) from Carrifran Wildwood, Scotland, used to generate Pacific Biosciences and 10X genomics data. ( **C**) Dorsal surface view of wings from specimen SC_VC_1220 (ilVanCard3) from Yellowcraig, Scotland, used to generate Hi-C data. ( **D**) Ventral surface view of wings from specimen SC_VC_1220 (ilVanCard3) from Yellowcraig, Scotland, used to generate Hi-C data.

The genome was sequenced from a single female *V. cardui* (ilVanCard2; Figure 1A, B) collected from Carrifran Wildwood, Scotland (latitude 55.400132, longitude -3.3352). Hi-C data were generated from a second female *V. cardui* (ilVanCard3; Figure 1C, D) collected from Yellowcraig, East Lothian, Scotland (latitude 56.062445, longitude -2.769836). A total of 25-fold coverage in Pacific Biosciences single-molecule long reads (N50 15 kb) and 89-fold coverage in 10X Genomics read clouds were generated. Primary assembly contigs were scaffolded with chromosome conformation Hi-C data. Manual assembly curation corrected 79 missing/misjoins and removed 7 haplotypic duplications, reducing the assembly size by 0.48% and scaffold number by 61.70%, and increasing the scaffold N50 by 21.74%.

**Figure 2.**
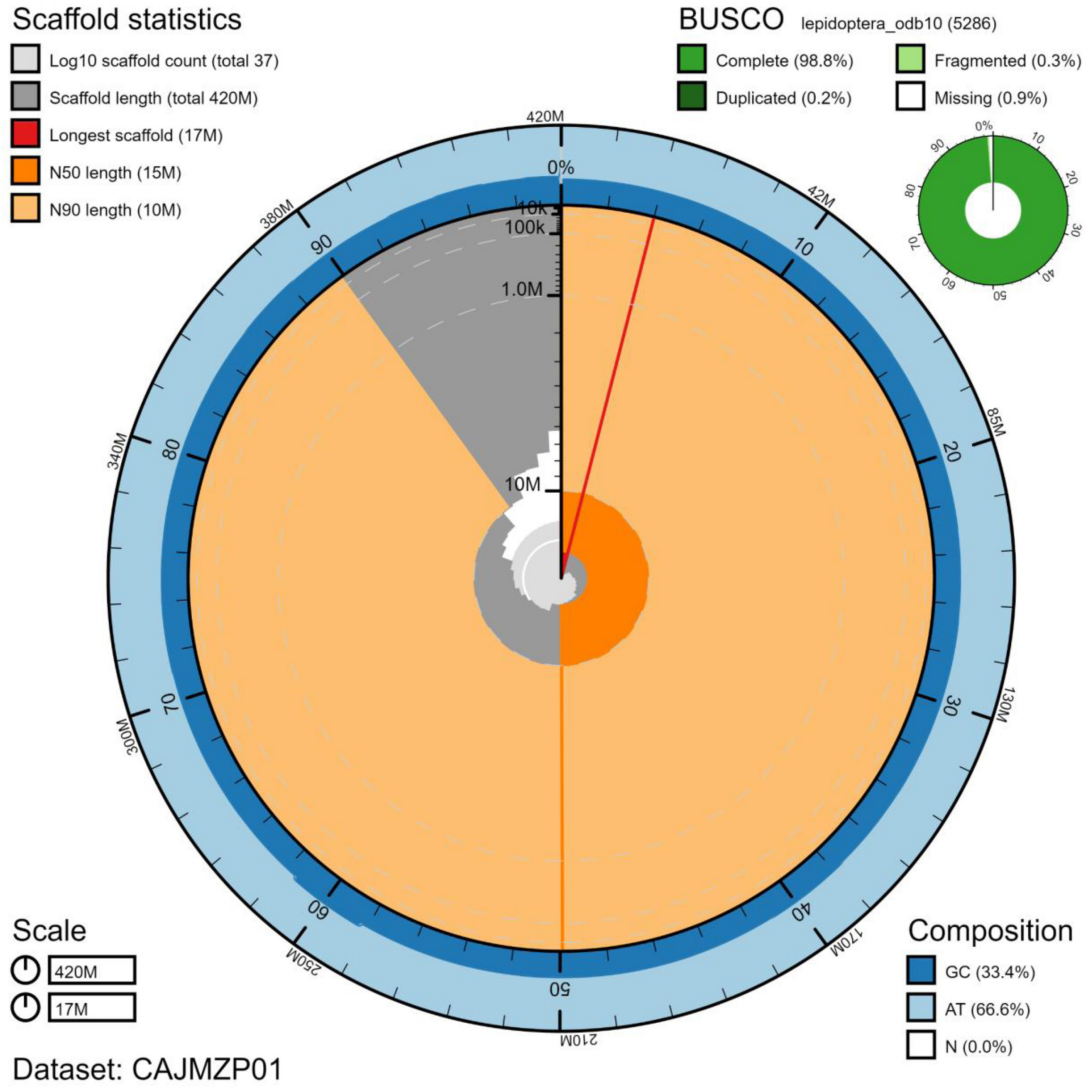
Genome assembly of *Vanessa cardui*, ilVanCard2.1: metrics. The BlobToolKit Snailplot shows N50 metrics and BUSCO gene completeness. The main plot is divided into 1,000 size-ordered bins around the circumference with each bin representing 0.1% of the 424,813,639 bp assembly. The distribution of chromosome lengths is shown in dark grey with the plot radius scaled to the longest chromosome present in the assembly (17,040,296 bp, shown in red). Orange and pale-orange arcs show the N50 and N90 chromosome lengths (14,615,999 and 9,960,137 bp), respectively. The pale grey spiral shows the cumulative chromosome count on a log scale with white scale lines showing successive orders of magnitude. The blue and pale-blue area around the outside of the plot shows the distribution of GC, AT and N percentages in the same bins as the inner plot. A summary of complete, fragmented, duplicated and missing BUSCO genes in the lepidoptera_odb10 set is shown in the top right. An interactive version of this figure is available at https://blobtoolkit.genomehubs.org/view/ilVanCard2.1/dataset/CAJMZP01/snail.

The final assembly has a total length of 425 Mb in 37 sequence scaffolds with a scaffold N50 of 15 Mb ( Table 1). Of the assembly sequence, 96.0% was assigned to 32 chromosomal-level scaffolds, representing 30 autosomes (numbered by sequence length), and the W and Z sex chromosome ( Figure 2– Figure 5; Table 2). The assembly has a BUSCO ( Simão *et al.*, 2015) completeness of 98.8% using the lepidoptera_odb10 reference set. While not fully phased, the assembly deposited is of one haplotype. Contigs corresponding to the second haplotype have also been deposited.

**Table 1.** Genome data for *Vanessa cardui*, ilVanCard2.1.

*Project accession data*	
Assembly identifier	ilVanCard2
Species	*Vanessa cardui*
Specimen	ilVanCard1 (RNA-Seq); ilVanCard2 (genome assembly); ilVanCard3 (Hi-C)
NCBI taxonomy ID	NCBI:txid171605
BioProject	PRJEB42869
BioSample ID	SAMEA7523147
Isolate information	Female, whole organisms
*Raw data accessions*	
PacificBiosciences SEQUEL II	ERR6608653
10X Genomics Illumina	ERR6054369-ERR6054372
Hi-C Illumina	ERR6054373
Illumina polyA RNA-Seq	ERR6054374
*Genome assembly*	
Assembly accession	GCA_905220365.1
*Accession of alternate haplotype*	GCA_905220355.1
Span (Mb)	425
Number of contigs	128
Contig N50 length (Mb)	7
Number of scaffolds	37
Scaffold N50 length (Mb)	15
Longest scaffold (Mb)	17
BUSCO * genome score	C:98.2%[S:97.9%,D:0.3%], F:0.8%,M:1.0%,n:1658
*Gene annotation*	
Number of protein coding genes	12,821
Average coding sequence length (bp)	1,738
Average number of exons per transcript	9.44
Average exon size (bp)	393
Average intron size (bp)	2358

* BUSCO scores based on the lepidoptera_odb10 BUSCO set using v5.1.2. C= complete [S= single copy, D=duplicated], F=fragmented, M=missing, n=number of orthologues in comparison. A full set of BUSCO scores is available at https://blobtoolkit.genomehubs.org/view/Vanessa%20cardui/dataset/CAJMZP01/busco.

**Figure 3.**
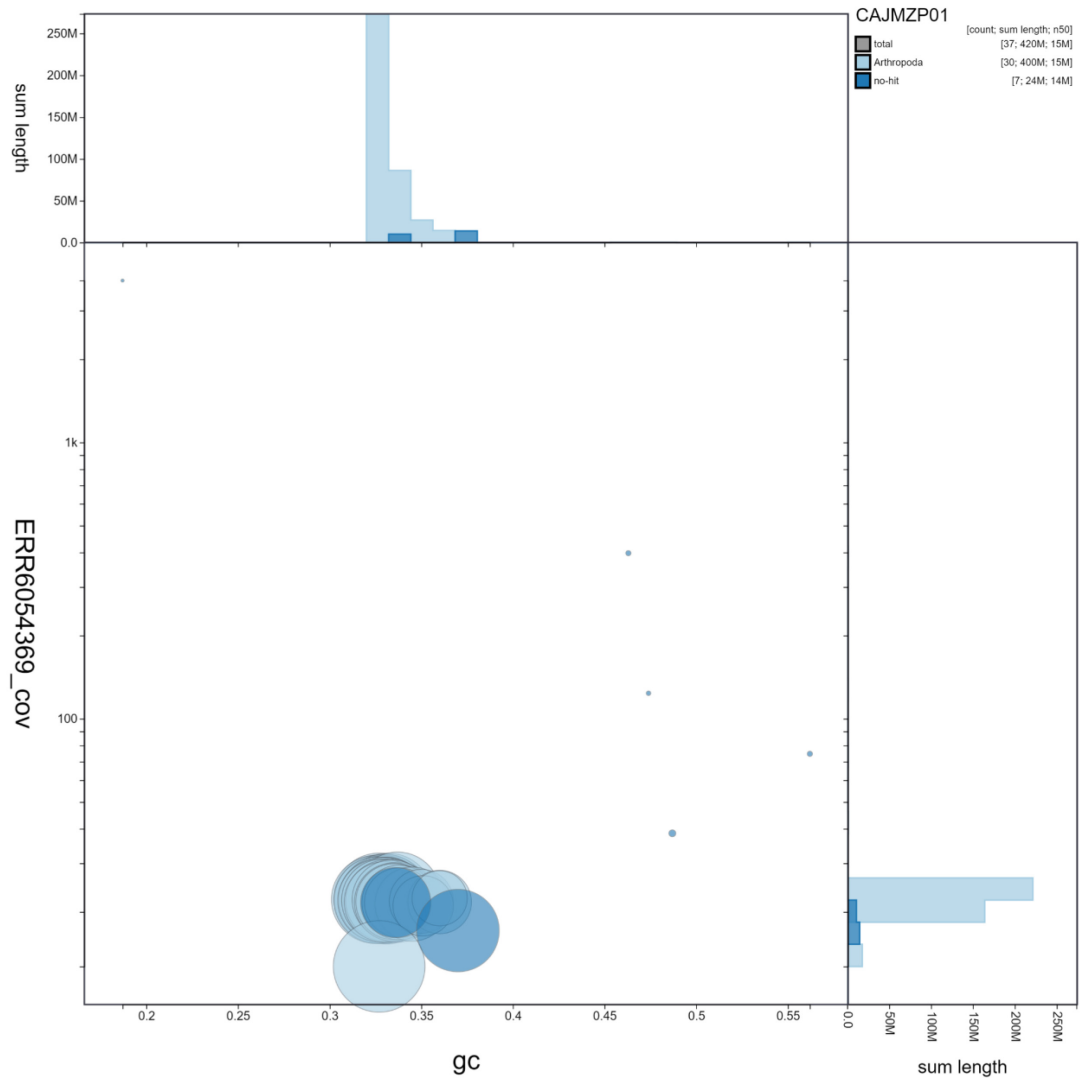
Genome assembly of *Vanessa cardui*, ilVanCard2.1: GC coverage. BlobToolKit GC-coverage plot. Scaffolds are coloured by phylum. Circles are sized in proportion to scaffold length. Histograms show the distribution of scaffold length sum along each axis. An interactive version of this figure is available at https://blobtoolkit.genomehubs.org/view/ilVanCard2.1/dataset/CAJMZP01/blob.

**Figure 4.**
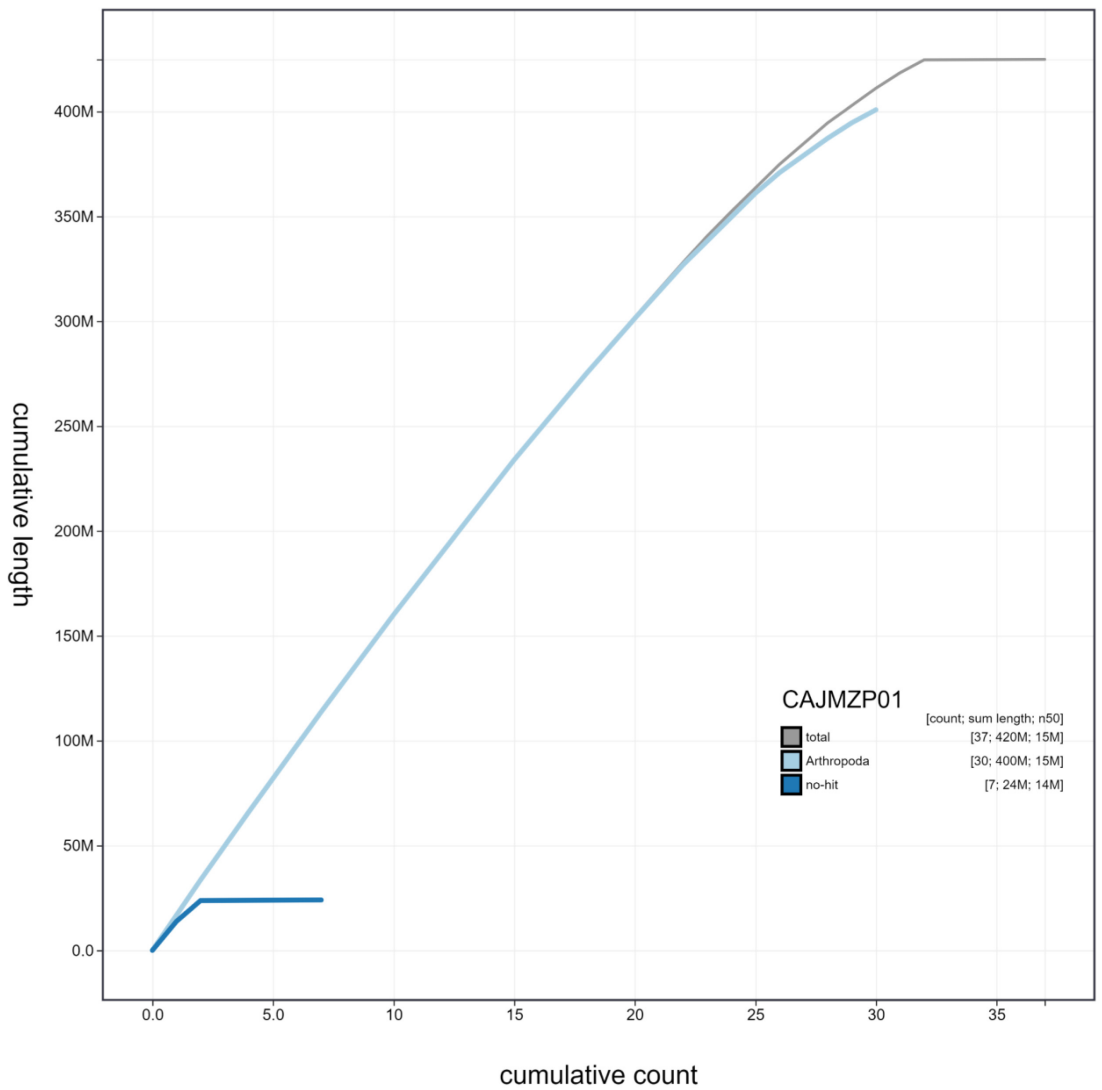
Genome assembly of *Vanessa cardui*, ilVanCard2.1: cumulative sequence. BlobToolKit cumulative sequence plot. The grey line shows cumulative length for all scaffolds. Coloured lines show cumulative lengths of scaffolds assigned to each phylum using the buscogenes taxrule. An interactive version of this figure is available at https://blobtoolkit.genomehubs.org/view/ilVanCard2.1/dataset/CAJMZP01/cumulative.

**Figure 5.**
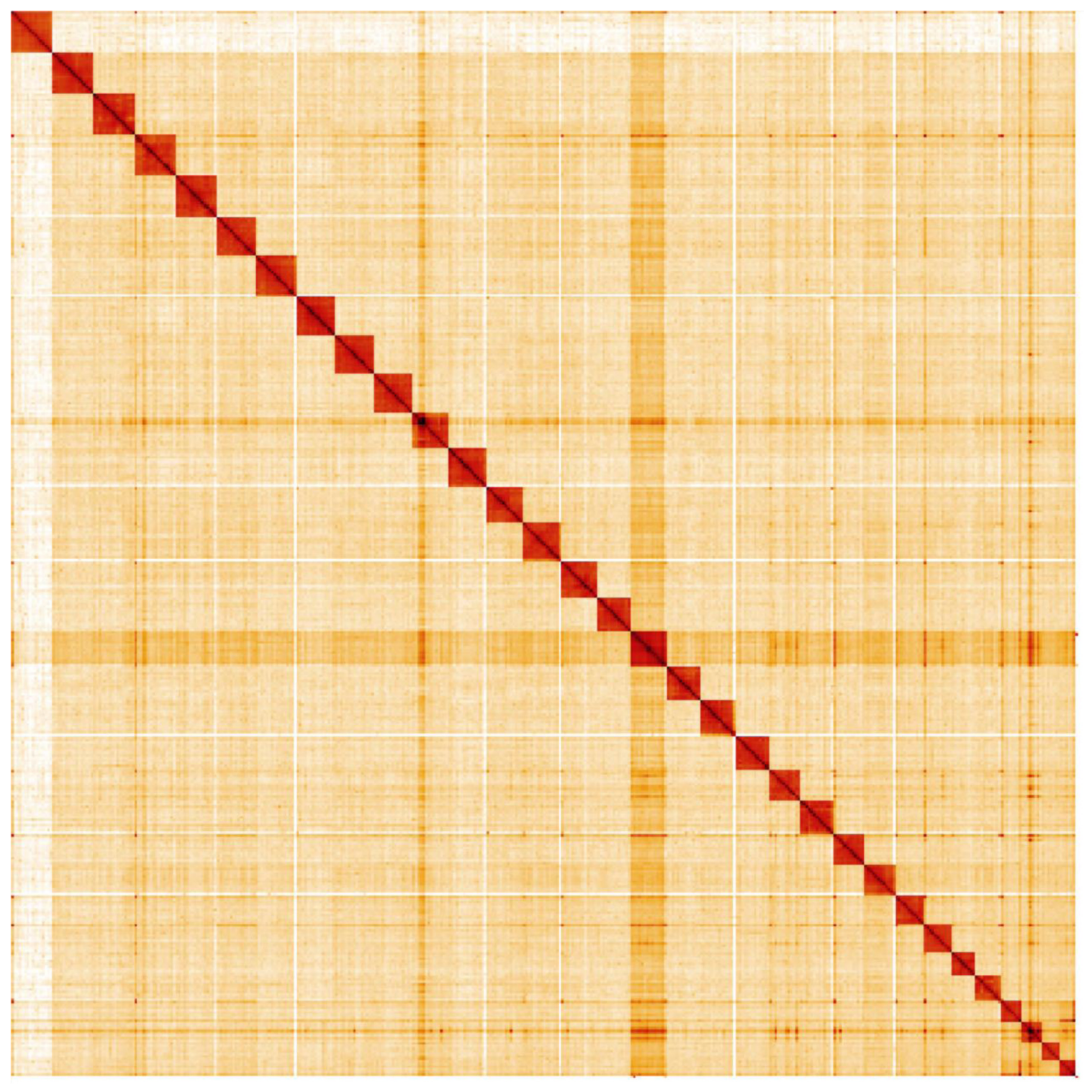
Genome assembly of *Vanessa cardui*, ilVanCard2.1: Hi-C contact map. Hi-C contact map of the ilVanCard2.1 assembly, visualised in HiGlass. Chromosomes are arranged by size from left to right and top to bottom.

**Table 2.** Chromosomal pseudomolecules in the genome assembly of *Vanessa cardui*, ilVanCard2.1.

INSDC accession	Chromosome	Size (Mb)	GC%
LR999925.1	1	16.61	32.7
LR999926.1	2	16.36	33.2
LR999927.1	3	16.09	33.1
LR999928.1	4	16.00	32.7
LR999929.1	5	15.95	32.9
LR999930.1	6	15.72	32.5
LR999931.1	7	15.57	33
LR999932.1	8	15.43	32.9
LR999933.1	9	15.30	32.6
LR999934.1	10	14.95	33.7
LR999935.1	11	14.87	33
LR999936.1	12	14.77	33.1
LR999937.1	13	14.62	33
LR999938.1	14	14.61	32.8
LR999939.1	15	13.92	32.7
LR999941.1	16	13.77	32.9
LR999942.1	17	13.55	33.1
LR999943.1	18	13.24	33
LR999944.1	19	12.93	33.7
LR999945.1	20	12.86	33.4
LR999946.1	21	12.59	33.5
LR999947.1	22	11.70	33.5
LR999948.1	23	11.33	33.8
LR999949.1	24	11.20	34.5
LR999950.1	25	9.96	33.6
LR999951.1	26	9.84	33.8
LR999952.1	27	8.26	35
LR999953.1	28	8.18	36
LR999954.1	29	7.38	35.1
LR999955.1	30	6.17	36
LR999940.1	W	13.82	37
LR999924.1	Z	17.04	32.7
LR999956.1	MT	0.02	19
-	Unplaced	0.22	49.5

## Gene annotation

The Ensembl gene annotation system ( Aken *et al.*, 2016) was used to generate annotation for the *Vanessa cardui* assembly (GCA_905220365.1, see https://rapid.ensembl.org/Vanessa_cardui_GCA_905220365.1/; Table 1). The annotation was created primarily through alignment of transcriptomic data to the genome, with gap filling via protein-to-genome alignments of a select set of proteins from UniProt ( UniProt Consortium, 2019) and OrthoDB ( Kriventseva *et al.*, 2008). Prediction tools, CPC2 ( Kang *et al.*, 2017) and RNAsamba ( Camargo *et al.*, 2020), were used to aid determination of protein coding genes.

## Methods

### Sample acquisition and nucleic acid extraction

The first female *V. cardui*, ilVanCard2 (genome assembly), was collected from Carrifran Wildwood, Scotland (latitude 55.400132, longitude -3.3352). Two further female *V. cardui* specimens, ilVanCard1 (RNA-Seq) and ilVanCard3 (Hi-C), was collected from Yellowcraig, East Lothian, Scotland (latitude 56.062445, longitude -2.769836). All samples were collected and identified by Konrad Lohse, University of Edinburgh, and were snap-frozen from life in liquid nitrogen.

DNA was extracted at the Wellcome Sanger Institute (WSI) Scientific Operations core from the whole organism using the Qiagen MagAttract HMW DNA kit, according to the manufacturer’s instructions. RNA was extracted in the Tree of Life Laboratory at the WSI using TRIzol (Invitrogen), according to the manufacturer’s instructions. RNA was then eluted in 50 μl RNAse-free water and its concentration RNA assessed using a Nanodrop spectrophotometer and Qubit Fluorometer using the Qubit RNA Broad-Range (BR) Assay kit. Analysis of the integrity of the RNA was done using Agilent RNA 6000 Pico Kit and Eukaryotic Total RNA assay.

### Sequencing

Pacific Biosciences HiFi circular consensus and 10X Genomics Chromium read cloud sequencing libraries were constructed according to the manufacturers’ instructions. Poly(A) RNA-Seq libraries were constructed using the NEB Ultra II RNA Library Prep kit. Sequencing was performed by the Scientific Operations core at the Wellcome Sanger Institute on Pacific Biosciences SEQUEL II (HiFi), Illumina HiSeq X (10X) and Illumina HiSeq 4000 (RNA-Seq) instruments. Hi-C data were generated using the Arima v1 Hi-C kit and sequenced on HiSeq X.

### Genome assembly

Assembly was carried out with Hifiasm ( Cheng *et al.*, 2021). Haplotypic duplication was identified and removed with purge_dups ( Guan *et al.*, 2020). One round of polishing was performed by aligning 10X Genomics read data to the assembly with longranger align, calling variants with freebayes ( Garrison & Marth, 2012). The assembly was then scaffolded with Hi-C data ( Rao *et al.*, 2014) using SALSA2 ( Ghurye *et al.*, 2019). The assembly was checked for contamination and corrected using the gEVAL system ( Chow *et al.*, 2016) as described previously ( Howe *et al.*, 2021). Manual curation was performed using gEVAL, HiGlass ( Kerpedjiev *et al.*, 2018) and Pretext. The mitochondrial genome was assembled using MitoHiFi ( Uliano-Silva *et al.*, 2021). The genome was analysed and BUSCO scores generated within the BlobToolKit environment ( Challis *et al.*, 2020). Table 3 contains a list of all software tool versions used, where appropriate.

**Table 3.** Software tools used.

Software tool	Version	Source
Hifiasm	0.12	Cheng *et al.*, 2021
purge_dups	1.2.3	Guan *et al.*, 2020
SALSA2	2.2	Ghurye *et al.*, 2019
longranger align	2.2.2	https://support.10xgenomics.com/genome-exome/ software/pipelines/latest/advanced/other-pipelines
freebayes	1.3.1-17- gaa2ace8	Garrison & Marth, 2012
MitoHiFi	1.0	Uliano-Silva *et al.*, 2021
gEVAL	N/A	Chow *et al.*, 2016
HiGlass	1.11.6	Kerpedjiev *et al.*, 2018
PretextView	0.1.x	https://github.com/wtsi-hpag/PretextView
BlobToolKit	2.6.2	Challis *et al.*, 2020

### Ethical/compliance issues

The materials that have contributed to this genome note were supplied by a Tree of Life collaborator. The Wellcome Sanger Institute employs a process whereby due diligence is carried out proportionate to the nature of the materials themselves, and the circumstances under which they have been/are to be collected and provided for use. The purpose of this is to address and mitigate any potential legal and/or ethical implications of receipt and use of the materials as part of the research project, and to ensure that in doing so we align with best practice wherever possible.

The overarching areas of consideration are:

- Ethical review of provenance and sourcing of the material;
- Legality of collection, transfer and use (national and international).

Each transfer of samples is undertaken according to a Research Collaboration Agreement or Material Transfer Agreement entered into by the Tree of Life collaborator, Genome Research Limited (operating as the Wellcome Sanger Institute) and in some circumstances other Tree of Life collaborators.

## Data availability

European Nucleotide Archive: Vanessa cardui (painted lady) genome assembly, ilVanCard2. Accession number PRJEB42869; https://identifiers.org/ena.embl/PRJEB42869.

The genome sequence is released openly for reuse. The *V. cardui* genome sequencing initiative is part of the Darwin Tree of Life (DToL) project. All raw sequence data and the assembly have been deposited in INSDC databases. Raw data and assembly accession identifiers are reported in Table 1.

## Author information

Members of the Darwin Tree of Life Barcoding collective are listed here: https://doi.org/10.5281/zenodo.4893704.

Members of the Wellcome Sanger Institute Tree of Life programme collective are listed here: https://doi.org/10.5281/zenodo.5377053.

Members of Wellcome Sanger Institute Scientific Operations: DNA Pipelines collective are listed here: https://doi.org/10.5281/zenodo.4790456.

Members of the Tree of Life Core Informatics collective are listed here: https://doi.org/10.5281/zenodo.5013542.

Members of the Darwin Tree of Life Consortium are listed here: https://doi.org/10.5281/zenodo.4783559.
