# Supplementary material for: The genome sequence of the painted lady, Vanessa cardui Linnaeus 1758
Source: Wellcome Open Res. 2021 Nov 26;6:324. [Version 1] doi: 10.12688/wellcomeopenres.17358.1 (PMC10061037; doi:10.12688/wellcomeopenres.17358.1)
Supplement: Peer review history

# Peer review: Reviewer response for version 1

**DOI:** 10.21956/wellcomeopenres.19192.r55528

**Published:** 2023-03-29

**Authors:** Nicholas VanKuren

**Affiliations:** 1 University of Chicago, Chicago, USA

Lohse and colleagues assembled and annotated the genome sequence of a cosmopolitan butterfly, *Vanessa cardui, *and present general statistics on its quality. WSI ToL has published many short reports like this recently, and this manuscript follows a similar format as those before. Despite redundancy with an available *V. cardui *sequence, this genome sequence and annotation are high-quality and will be of interest to butterfly genomics, population genomics, and genetics researchers. 

Very minor comments:

- Fig. 1: It would be nice to note that these are all females in the figure, or at least in the legend.
- Results: BUSCO completeness in text and Fig. 2 listed as 98.8%, but Table 1 shows 98.2%.
- Fig. 5: it would be nice to point out the W and Z here so that people can immediately visualize. 
- Gene annotation: Perhaps this has been listed in previous publications, but what is the "select set of proteins from UniProt and OrthoDB"? This would be useful to know for other groups looking to annotate their genomes. In general, there should be a section on gene annotation in the Methods giving a few more details on important parameters used for predictions.
- Methods: "Two further female ... was" --> "were".

Are sufficient details of methods and materials provided to allow replication by others?

Partly

Is the rationale for creating the dataset(s) clearly described?

Yes

Are the datasets clearly presented in a useable and accessible format?

Yes

Are the protocols appropriate and is the work technically sound?

Yes

Reviewer Expertise:

Genetics, genomics, developmental biology

I confirm that I have read this submission and believe that I have an appropriate level of expertise to confirm that it is of an acceptable scientific standard.

# Peer review: Reviewer response for version 1

**DOI:** 10.21956/wellcomeopenres.19192.r54282

**Published:** 2023-01-31

**Authors:** Li-Wei Wu

**Affiliations:** 1 Tunghai University,, Taichung,, Taiwan

The presented manuscript entitled "The genome sequence of the painted lady, *Vanessa cardui *Linnaeus 1758" has been evaluated. The writing and flow are concise and clear to public readers. The provided data could be useful for further work. As a reader, I am happy to read this article. Only some suggestions are provided as follows, and most of them are pointing out that the association between used sample and its produced sequence data should be clear. For example, I cannot find out which individual produced the RNA-seq data.

 Some suggestions are as following:

"Abstract" section:

- The presented genome of *Vanessa cardui* seems like its from two female individuals, please revise it.

"Genome sequence report" section:

- "The genome was sequenced from a single female": need to revise. More than one sample was detected in the text.

"Methods" section:

 "sample acquisition and nucleic acid extraction" part:

- There are three individuals in this genome assembling, please check all the used materials and their associated datasets.
- RNA-seq data was from which individual? The collection data should be provided.

"Genome assembly" part:

- The authors have assembled mitochondrial genome, but no associated accession number is provided.

Discussion (if possible):

 The authors have noticed Zhang *et al.* (2021) published another high-quality genome of Vanessa cardui, and the authors presented the second report to this organism. I think the authors should give some discussion between these two works, making readers aware what you find when a new genome is generated. For example, the two works present different protein-coding genes in the same species, why?

Are sufficient details of methods and materials provided to allow replication by others?

Partly

Is the rationale for creating the dataset(s) clearly described?

Yes

Are the datasets clearly presented in a useable and accessible format?

Yes

Are the protocols appropriate and is the work technically sound?

Yes

Reviewer Expertise:

molecular phylogeny, historical biogeography, genome from museum specimen, mitochondrial genome

I confirm that I have read this submission and believe that I have an appropriate level of expertise to confirm that it is of an acceptable scientific standard.
